# Glaucomatous Optic Neuropathy Management: the Role of Neuroprotective Agents

**Published:** 2013

**Authors:** Marianne L. Shahsuvaryan

**Affiliations:** Yerevan State Medical University, Yerevan, Armenia

**Keywords:** Glaucoma, Ganglion cell neurodegeneration, Neuroprotection, Calcium channel blockers

## Abstract

Glaucoma is a major cause of worldwide irreversible blindness. The central role of raised intraocular pressure (IOP) is being questioned as many patients continue to demonstrate a clinically downhill course despite initial control of IOP. The latest concept of recognizing glaucoma as a multifactorial, progressive, neurodegenerative disease of retinal ganglion cells (RGCSs) associated with characteristic axon degeneration in the optic nerve emphasizes that several pressure-independent mechanisms are responsible for the development and progression of glaucomatous optic neuropathy. Neuroprotection as a pharmacological strategy to mitigate retinal ganglion cell death has been a popular current approach. The aim of this review is to evaluate the neuroprotective potential of calcium channel blockers in glaucomatous optic neuropathy.

## INTRODUCTION

Glaucoma has been considered as a major cause of worldwide irreversible blindness [1]. About 66.8 million people worldwide are afflicted with glaucoma [2]. Glaucoma is currently recognized to be a multifactorial, progressive neurodegenerative disorder. It is characterized by the acquired death of retina ganglion cells (RGCSs) and loss of their axons as well as optic nerve atrophy and loss of neurons in the lateral geniculate nucleus and the visual cortex [3]. This concept emphasizes that several pressure-independent mechanisms are responsible for the development and progression of glaucomatous neuropathy and that high intra-ocular pressure (IOP) and vascular insufficiency in the optic nerve head are merely risk factors for the development of glaucoma. The central role of raised IOP is being questioned as many patients continue to demonstrate a clinically downhill course despite initial control of raised IOP [4]. In addition, up to one-sixth of patients with glaucoma develop it despite normal IOP [5]. Chronic heart failure is associated with lower ocular perfusion pressure, and glaucomatous optic nerve head changes [6]. Visual field loss and RGCs death continue to occur in patients with well controlled intraocular pressures and thus, a consensus has recently been reached that additional treatment strategies are needed [7]. 

In humans, the optic nerve consists of approximately one million axons; with the majority of the cell bodies are primarily located in the ganglion cell layer [8]. RGCs death, therefore, represents the final common pathway of virtually all diseases of the optic nerve including glaucomatous optic neuropathy. There is histological and electrophysiological evidence to suggest that ganglion cells are the sole neurons affected in glaucoma [8]. All animal cells are programmed for carrying out self-destruction when they are not needed, or when damaged. Apoptosis is a process rather than an event. It has been labeled a programmed cell death, or cell suicide. It is not unique to RGCSs or glaucoma alone. Following an initial insult, the cells try to minimize or buffer the damage done through a variety of processes. Generation of “suicide triggers” could be one of the consequences of these processes and interactions and these molecules may start the process of apoptosis which is characterized by an orderly pattern of inter-nucleosomal DNA fragmentation, chromosome clumping, cell shrinkage and membrane blebbing [9]. Abnormally high calcium ion concentration leads to inappropriate activation of complex cascades of nucleases, proteases and lipases. They directly attack cell constituents and lead to the generation of highly reactive free radicals and activation of the nitric oxide pathway [10]. The resulting interaction between intermediate compounds and free radicals leads to DNA nitrosylation, fragmentation and activation of the apoptotic program. The major causes for cell death following activation of NMDA (N-methyl-D-aspartate) receptors are the influx of calcium and sodium into cells, the generation of free radicals linked to the formation of advanced glycation endproducts (AGEs) and/or advanced lipoxidation endproducts (ALEs) as well as defects in the mitochondrial respiratory chain [11,12]. The general consensus is that intracellular concentrations of calcium ion are increased in apoptosis [13-16]. An increase in intracellular calcium is neurotoxic through activation of calcium-dependent catabolic enzymes [17]. 

The idea of treating glaucoma with neuroprotection goes back to the 1990s, with Weinreb and Levin [18] writing in Archives of Ophthalmology that, at the very least, neuroprotection should be an adjunctive therapy, along with lowering IOP. Neuroprotection and possibly neuroregeneration and neuro-enhancement would be future treatment modality [19]. The concept of neuroprotective therapy for glaucoma is that damage to retinal ganglion cells may be prevented by intervening in neuronal death pathways [20-22]. The right pharmacologic agent with a meaningful intraocular penetration would rationalize the neuroprotection strategy in glaucoma [23]. Calcium ion dependent intracellular mechanisms related to glaucoma were recently reviewed by Crish and Calkins [24]. Calcium channel blockers have been shown to neutralize glutamate-NMDA-induced intracellular calcium ion influx. Neuroprotective effect of calcium channel blockers against retinal ganglion cell damage under hypoxia was shown by Yamada et al. [25], and also by Garcia-Campos et al. [26].

## HYPOTHESIS

Understanding of the role of extracellular calcium transport across cell membranes in modulating various intracellular signaling processes, including the initiation of the apoptotic cascade, represents the rationale for interest in investigating calcium-channel blockers for neuroprotection in glaucoma. Calcium channel blockers may potentially inhibit ganglion cells and photoreceptor apoptosis in glaucoma representing a viable option for glaucomatous optic neuropathy management.

## DISCUSSION

Vascular dysregulation has been implicated in primary open-angle glaucoma. One theory is that ischemia of the retina is caused by lack of adequate blood supply due to the squeeze experienced by the blood vessels serving the optic nerve and the retina as a result of the high IOP. This squeeze can also cause blood vessels to burst, resulting in hemorrhage. Calcium channel blockers are known to relieve the pressure on the blood vessels [27].

Calcium channel blockers generally dilate isolated ocular vessels and increase ocular blood flow in experimental animals, normal humans, and patients with open-angle glaucoma and in patients who have vascular diseases in which considerable vascular tone is present [28-30]. As well, contrast sensitivity in patients with normal tension glaucoma was found ameliorated by calcium channel inhibition [31-32]. 

In a retrospective study of normal-tension and open-angle glaucoma patients who happened to be taking calcium channel blockers, Netland et al. [33] demonstrated a decrease in glaucoma progression relative to controls. Otori et al. [34] evaluated the effect of diltiazem on inhibition of glutamate-induced apoptotic retinal ganglion cell death and concluded that application of diltiazem does not appear to reduce apoptosis.

Nimodipine is an isopropyl calcium channel blocker which readily crosses the blood-brain barrier due to its high lipid solubility. Its primary action is to reduce the number of open calcium channels in cell membranes, thus restricting influx of calcium ions into cells.

Yamada et al. [25] in an experimental in vitro model revealed that nimodipine has a direct neuroprotective effect against retinal ganglion cells damage related to hypoxia. Michelson et al. [35] have evaluated the impact of nimodipine on retinal blood flow in double-blind, two-way, crossover study of healthy subjects and found that orally administered at a dosage of 30 mg three times a day nimodipine significantly increases retinal perfusion in healthy subjects. 

The impact of nimodipine on ocular circulation in normal tension glaucoma has been evaluated in many clinical studies. Piltz et al. [36] have described a performance-corrected improvement in visual field deviation and contrast sensitivity in patients with normal tension glaucoma (NTG) and in control subjects in a prospective, placebo-controlled double-masked study after oral administration of nimodipine (30 mg twice a day). Wang et al. [37] also stated that oral administration of nimodipine is useful for improvement of visual field defect in patients with normal-tension glaucoma. Other authors [38] also evidenced that a single dose of 30mg nimodipine normalizes the significantly reduced retinal blood flow in NTG patients with clinical signs of vasospasmic hyperactivity. Luksch et al. [29] have examined the impact of 60 mg nimodipine in NTG patients two hours following oral administration. Results disclosed that nimodipine increased the blood flow of the optic nerve head by 18% and improved color-contrast sensitivity. Thus, nimodipine is potentially useful calcium channel blocker for eye disorders treatment due to its high lipid solubility and ability to cross the blood-brain barrier. 

Recent experimental evidence suggests that Nilvadipine appears to have beneficial effects on different ocular structures. Ogata et al. [39] have evaluated the effects of nilvadipine on retinal blood flow and concluded that this agent may directly and selectively increase retinal tissue blood flow, while having only minimal effect on systemic circulation including arterial blood pressure. Another experimental study conducted by Uemura and Mizota [40] have also advocated the use of nilvadipine for the treatment of glaucoma or other retinal diseases that have some relation to apoptosis, based on claims that nilvadipine has high permeability to retina and neuroprotective effect to retinal cells. Otori et al. [34] in the experimental study of different calcium channel blockers protective effect against glutamate neurotoxicity in purified retinal ganglion cells has found that nilvadipine significantly reduces glutamate-induced apoptosis. In addition to the direct effects of calcium channel blockers on intracellular concentrations of calcium ion in ganglion cells, another indirect effect is expected such as increased choroidal blood flow [30]. Several clinical trials have shown the effectiveness of nilvadipine in glaucoma. 

Yamamoto et al. [41], Tomita et al. [42], Niwa et al. [43] have found that nilvadipine reduces vascular resistance in distal retrobulbar arteries and significantly increases velocity in the central retinal artery in patients with normal tension glaucoma. Tomita et al. [43] also stated that reduced orbital vascular resistance after a 4-week treatment with 2 mg oral nilvadipine consequently increases the optic disc blood flow. Koseki et al. [30] conducted a randomized, placebo-controlled, double-masked, single-center 3-year study of nilvadipine on visual field and ocular circulation in glaucoma with low-normal pressure. No topical ocular hypotensive drugs were prescribed. The authors concluded that nilvadipine (2 mg twice daily) slightly slowed the visual field progression and maintained the optic disc rim, and the posterior choroidal circulation increased over 3 years in patients with open-angle glaucoma with low–normal intraocular pressure. 

In the latest clinical trial, Araie [44] showed evidence that a low dose of oral nilvadipine (4mg/day) significantly decreased the rate of mean deviation deterioration in NTG patients as compared to placebo. The results of these studies add to the growing body of evidence that nilvadipine may be useful for neuroprotection in glaucoma. 

Thus, nilvadipine can alleviate damage at various levels of visual pathway [44] and is a potentially useful calcium channel blocker for eye disorders treatment due to its hydrophobic nature with high permeability to the central nervous system, including the retina and has the highest antioxidant potency among calcium channel blockers.

Flunarizine, a potent calcium channel blocker has been demonstrated to enhance RGCs survival after optic nerve transection in mice [45]. It should be recognized that the neuroprotective action of flunarizine may occur partly because of increased blood flow to the affected tissues. Flunarizine is known to have vasodilatory properties and to improve blood flow to the optic nerve head in patients with low-tension glaucoma [46]. Flunarizine itself has been demonstrated to lower IOP in the monkey eye when applied topically [47]. This finding not only confirms the usefulness of calcium channel blockers as neuroprotectants, but also shows that they can be effective when topically applied, at least in the case of flunarizine. Such data support the idea that when topically applied to the human eye, flunarizine may promote survival of some retinal ganglion cells in glaucoma [48]. 

A neuroprotective effect of another new calcium channel blocker–Lomerizine have been evaluated in many experimental and clinical studies [49-52].

Tamaki et al. [49] investigated the effects of lomerizine on the ocular tissue circulation in rabbits and on the circulation in the optic nerve head and choroid in healthy volunteers and have found that lomerizine increases blood velocity, and probably blood flow, in the optic nerve head and retina in rabbits, and it also increases blood velocity in the optic nerve head in healthy humans, without significantly altering blood pressure or heart rate. Evidence from the study conducted by Hara et al. [50] also suggested that in healthy humans, lomerizine increased blood velocity in the optic nerve head, without significantly altering blood pressure or heart rate. Moreover, lomerizine reduced retinal damage in rats both in vitro and in vivo, presumably through a calcium channel blocking effect via an action that may involve a direct protection of retinal neurons as well as an improvement in the ocular circulation. These results indicate that lomerizine may be useful as a therapeutic drug against ischemic retinal diseases (such as glaucoma and retinal vascular occlusive diseases) that involve a disturbance of the ocular circulation. The general consensus is that lomerizine do not seem to affect systemic hypotension [49-51]. 

In an experimental study, Karim et al. [53] have found that lomerizine alleviates secondary degeneration of retinal ganglion cells induced by an optic nerve crush injury in the rat, presumably by improving the impaired axoplasmic flow. The latest experimental study [3] evaluated protective properties against neuronal degeneration within the dorsal lateral geniculate nucleus and superior colliculus in mice eyes evidenced that lomerizine reduces the retinal damage and affords some protection against transsynaptic neuronal degeneration within the visual center of the mouse brain.

At present Santen Pharmaceutical has lomerizine in Phase II trials to inhibit the progression of visual field defects [54]. Lomerizine and nilvadipine significantly increased optic nerve head tissue blood velocity in the untreated normal monkey eyes, while significant, less of an increase was found in the experimental glaucomatous eyes [55].

## CONCLUSION

Currently, glaucoma is recognized as a multifactorial, progressive, neurodegenerative disorder and is characterized by the acquired death of retina ganglion cells and loss of their axons as well as optic nerve atrophy and loss of neurons in the lateral geniculate nucleus and the visual cortex. Neuroprotection as a pharmacological strategy to shield retinal ganglion cell death has been a popular approach. 

Current studies show that neuroprotection and possibly neuroregeneration and neuro-enhancement would be the future treatment modality. Calcium channel blockers may potentially inhibit ganglion cells and photoreceptor apoptosis in glaucoma representing a viable option for glaucomatous optic neuropathy management. The proposed new drugs have shown good promise. The most suitable calcium channel blocker with a meaningful intraocular penetration would rationalize the neuroprotection strategy in glaucoma. 

## DISCLOSURE

Conflicts of Interest: None declared.

## References

[1] Quigley HA, Broman AT (2006). The number of people with glaucoma worldwide in 2010 and 2020. Br J Ophthalmol.

[2] Vyas P, Naik U, Gangaiah JB (2011). Efficacy of bimatoprost 0.03% in reducing intraocular pressure in patients with 360° synechial angle-closure glaucoma:a Preliminary study. Indian J Ophthalmol.

[3] Kaushik S, Pandav SS, Ram J (2003). Neuroprotection in glaucoma. J Postgrad Med.

[4] Osborne NN, Ugarte M, Chao M, Chidlow G, Bae JH, Wood JP, Nash MS (1999). Neuroprotection in relation to retinal ischemia and relevance to glaucoma. Surv Ophthalmol.

[5] Brubaker RF (1996). Delayed functional loss in glaucoma. LII Edward Jackson Memorial Lecture. Am J Ophthalmol.

[6] Meira-Freitas D, Melo LA Jr, Almeida-Freitas DB, Paranhos A Jr (2012). Glaucomatous optic nerve head alterations in patients with chronic heart failure. Clin Ophthalmol.

[7] Chidlow G, Wood JP, Casson RJ (2007). Pharmacological neuroprotection for glaucoma. Drugs.

[8] Liesegang TJ (1996). Glaucoma: changing concepts and future directions. Mayo Clin Proc.

[9] Farkas RH, Grosskreutz CL (2001). Apoptosis, neuroprotection, and retinal ganglion cell death: an overview. Int Ophthalmol Clin.

[10] Naskar R, Dreyer EB (2001). New horizons in neuroprotection. Surv Ophthalmol.

[11] Schmidt KG, Bergert H, Funk RH (2008). Neurodegenerative diseases of the retina and potential for protection and recovery. Curr Neuropharmacol.

[12] Hayashi H, Eguchi Y, Fukuchi-Nakaishi Y, Takeya M, Nakagata N, Tanaka K, Vance JE, Tanihara H (2012). A potential neuroprotective role of apolipoprotein E-containing lipoproteins through low density lipoprotein receptor-related protein 1 in normal tension glaucoma. J Biol Chem.

[13] Nicotera P, Orrenius S (1998). The role of calcium in apoptosis. Cell Calcium.

[14] Delyfer MN, Léveillard T, Mohand-Saïd S, Hicks D, Picaud S, Sahel JA (2004). Inherited retinal degenerations: therapeutic prospects. Biol Cell.

[15] Sanges D, Comitato A, Tammaro R, Marigo V (2006). Apoptosis in retinal degeneration involves cross-talk between apoptosis-inducing factor (AIF) and caspase-12 and is blocked by calpain inhibitors. Proc Natl Acad Sci U S A.

[16] Paquet-Durand F, Johnson L, Ekström P (2007). Calpain activity in retinal degeneration. J Neurosci Res.

[17] Chua B, Goldberg I (2010). Neuroprotective agents in Glaucoma therapy: Recent developments and future Directions. Expert Rev Ophthalmol.

[18] Weinreb RN, Levin LA (1999). Is neuroprotection a viable therapy for glaucoma?. Arch Ophthalmol.

[19] Mowatt L, Mc Intosh M, Rumelt S "Strategies for Neuroprotection in Glaucoma.". Glaucoma - Basic and Clinical Aspects.

[20] Danesh-Meyer HV (2011). Neuroprotection in glaucoma: recent and future directions. Curr Opin Ophthalmol.

[21] Chang EE, Goldberg JL (2012). Glaucoma 2.0: neuroprotection, neuroregeneration, neuroenhancement. Ophthalmology.

[22] Sena DF, Ramchand K, Lindsley K (2010). Neuroprotection for treatment of glaucoma in adults. Cochrane Database Syst Rev.

[23] Velpandian T (2010). Closed gateways--can neuroprotectants shield the retina in glaucoma?. Drugs R D.

[24] Crish SD, Calkins DJ (2011). Neurodegeneration in glaucoma: progression and calcium-dependent intracellular mechanisms. Neuroscience.

[25] Yamada H, Chen YN, Aihara M, Araie M (2006). Neuroprotective effect of calcium channel blocker against retinal ganglion cell damage under hypoxia. Brain Res.

[26] García-Campos J, Villena A, Díaz F, Vidal L, Moreno M, Pérez de Vargas I (2007). Morphological and functional changes in experimental ocular hypertension and role of neuroprotective drugs. Histol Histopathol.

[27] Beidoe G, Mousa SA (2012). Current primary open-angle glaucoma treatments and future directions. Clin Ophthalmol.

[28] Araie M, Mayama C (2011). Use of calcium channel blockers for glaucoma. Prog Retin Eye Res.

[29] Luksch A, Rainer G, Koyuncu D, Ehrlich P, Maca T, Gschwandtner ME, Vass C, Schmetterer L (2005). Effect of nimodipine on ocular blood flow and colour contrast sensitivity in patients with normal tension glaucoma. Br J Ophthalmol.

[30] Koseki N, Araie M, Tomidokoro A, Nagahara M, Hasegawa T, Tamaki Y, Yamamoto S (2008). A placebo-controlled 3-year study of a calcium blocker on visual field and ocular circulation in glaucoma with low-normal pressure. Ophthalmology.

[31] Yu DY, Cringle S, Valter K, Walsh N, Lee D, Stone J (2004). Photoreceptor death, trophic factor expression, retinal oxygen status, and photoreceptor function in the P23H rat. Invest Ophthalmol Vis Sci.

[32] Boehm AG, Breidenbach KA, Pillunat LE, Bernd AS, Mueller MF, Koeller AU (2003). Visual function and perfusion of the optic nerve head after application of centrally acting calcium-channel blockers. Graefes Arch Clin Exp Ophthalmol.

[33] Netland PA, Chaturvedi N, Dreyer EB (1993). Calcium channel blockers in the management of low-tension and open-angle glaucoma. Am J Ophthalmol.

[34] Otori Y, Kusaka S, Kawasaki A, Morimura H, Miki A, Tano Y (2003). Protective effect of nilvadipine against glutamate neurotoxicity in purified retinal ganglion cells. Brain Res.

[35] Michelson G, Wärntges S, Leidig S, Lötsch J, Geisslinger G (2006). Nimodipine plasma concentration and retinal blood flow in healthy subjects. Invest Ophthalmol Vis Sci.

[36] Piltz JR, Bose S, Lanchoney D (1998). The effect of nimodipine, a centrally active calcium antagonist, on visual function and mascular blood flow in patients with normal-tension glaucoma and control subjects. J Glaucoma.

[37] Wang W, Shen N, Wang Y (2002). Effect of nimodipine on normal-tension glaucoma. Inter J Ophthalmol.

[38] Michalk F, Michelson G, Harazny J, Werner U, Daniel WG, Werner D (2004). Single-dose nimodipine normalizes impaired retinal circulation in normal tension glaucoma. J Glaucoma.

[39] Ogata Y, Kaneko T, Kayama N, Ueno S (2000). [Effects of nilvadipine on retinal microcirculation and systemic circulation]. Nihon Ganka Gakkai Zasshi.

[40] Uemura A, Mizota A (2008). Retinal concentration and protective effect against retinal ischemia of nilvadipine in rats. Eur J Ophthalmol.

[41] Yamamoto T, Niwa Y, Kawakami H, Kitazawa Y (1998). The effect of nilvadipine, a calcium-channel blocker, on the hemodynamics of retrobulbar vessels in normal-tension glaucoma. J Glaucoma.

[42] Tomita G, Niwa Y, Shinohara H, Hayashi N, Yamamoto T, Kitazawa Y (1999). Changes in optic nerve head blood flow and retrobular hemodynamics following calcium-channel blocker treatment of normal-tension glaucoma. Int Ophthalmol.

[43] Niwa Y, Yamamoto T, Harris A, Kagemann L, Kawakami H, Kitazawa Y (2000). Relationship between the effect of carbon dioxide inhalation or nilvadipine on orbital blood flow in normal-tension glaucoma. J Glaucoma.

[44] Araie M Neuroprotective therapy of the visual pathway in glaucoma. Acta Ophthalmologica.

[45] Eschweiler GW, Bähr M (1993). Flunarizine enhances rat retinal ganglion cell survival after axotomy. J Neurol Sci.

[46] Cellini M, Possati GL, Caramazza N, Profazio V, Caramazza R (1997). The use of flunarizine in the management of low-tension glaucoma: a color Doppler study. Acta Ophthalmol Scand Suppl.

[47] Siegner SW, Netland PA, Schroeder A, Erickson KA (2000). Effect of calcium channel blockers alone and in combination with antiglaucoma medications on intraocular pressure in the primate eye. J Glaucoma.

[48] Osborne NN, Wood JP, Cupido A, Melena J, Chidlow G (2002). Topical flunarizine reduces IOP and protects the retina against ischemia-excitotoxicity. Invest Ophthalmol Vis Sci.

[49] Tamaki Y, Araie M, Fukaya Y, Nagahara M, Imamura A, Honda M, Obata R, Tomita K (2003). Effects of lomerizine, a calcium channel antagonist, on retinal and optic nerve head circulation in rabbits and humans. Invest Ophthalmol Vis Sci.

[50] Hara H, Toriu N, Shimazawa M (2004). Clinical potential of lomerizine, a Ca2+ channel blocker as an anti-glaucoma drug: effects on ocular circulation and retinal neuronal damage. Cardiovasc Drug Rev.

[51] Muñoz-Negrete FJ, Pérez-López M, Won Kim HR, Rebolleda G (2009). [New developments in glaucoma medical treatment]. Arch Soc Esp Oftalmol.

[52] Ito Y, Nakamura S, Tanaka H, Tsuruma K, Shimazawa M, Araie M, Hara H (2010). Lomerizine, a Ca2+ channel blocker, protects against neuronal degeneration within the visual center of the brain after retinal damage in mice. CNS Neurosci Ther.

[53] Karim Z, Sawada A, Kawakami H, Yamamoto T, Taniguchi T (2006). A new calcium channel antagonist, lomerizine, alleviates secondary retinal ganglion cell death after optic nerve injury in the rat. Curr Eye Res.

[54] Dutton G (2012). Ocular Therapeutics Target the Retina. Wall Street BioBeat.

[55] Mayama C, Ishii K, Ota T, Tomidokoro A, Araie M (2012). Changes in optic nerve head circulation in response to vasoactive agents: intereye comparison in monkeys with experimental unilateral glaucoma. Invest Ophthalmol Vis Sci.

